# Mode-Dependent Effect of Xenon Inhalation on Kainic Acid-Induced Status Epilepticus in Rats

**DOI:** 10.3389/fncel.2019.00375

**Published:** 2019-08-14

**Authors:** Yurong Zhang, Mengdi Zhang, Jie Yu, Wei Zhu, Qiaoyun Wang, Xiaohong Pan, Xue Gao, Jing Yang, Hongliu Sun

**Affiliations:** ^1^School of Pharmaceutical Sciences, Binzhou Medical University, Yantai, China; ^2^Shandong Academy of Medical Sciences, Jinan, China

**Keywords:** status epilepticus, xenon, neuronal injury, kainic acid, seizure

## Abstract

Previous studies have reported the possible neuroprotective effects of xenon treatment. The purpose of this study was to define the range of effective xenon ratio, most effective xenon ratio, and time-window for intervention in the kainic acid (KA) – induced status epilepticus (SE) rat model. Different ratios of xenon (35% xenon, 21% oxygen, 44% nitrogen, 50% xenon, 21% oxygen, 29% nitrogen, 70% xenon, 21% oxygen, and 9% nitrogen) were used to treat the KA-induced SE. Our results confirmed the anti-seizure role of 50 and 70% xenon mixture, with a stronger effect from the latter. Further, 70% xenon mixture was dispensed at three time points (0 min, 15 min delayed, and 30 min delayed) after KA administration, and the results indicated the anti-seizure effect at all treated time points. The results also established that the neuronal injury in the hippocampus and entorhinal cortex (EC), assessed using Fluoro-Jade B (FJB) staining, were reversed by the xenon inhalation, and within 30 min after KA administration. Our study, therefore, indicates the appropriate effective xenon ratio and time-window for intervention that can depress seizures. The prevention of neuronal injury and further reversal of the loss of effective control of depress network in the hippocampus and EC may be the mechanisms underlying the anti-seizure effect of xenon.

## Introduction

Currently available anti-epileptic interventions such as anti-epileptic drugs, resection, and deep brain stimulation have a limited efficacy (Wang et al., 2015; Falcicchia et al., 2018; Kaur et al., 2019). Most patients with epilepsy depend on anti-epileptic drugs to control seizures. However, these possess a few drawbacks such as drug resistance that influence about one-third of the individuals (Schmidt and Löscher, 2005). The surgical treatment of epilepsy is not suitable for all refractory epilepsy cases (Wiebe et al., 2001; McIntosh et al., 2004). As the recently established epileptic therapy, deep brain stimulation could control a part of refractory epilepsy cases; however, it has a few drawbacks such as selection of stimulation region and parameters for different types of epilepsy (Cohen-Gadol et al., 2003; Theodore and Fisher, 2004) and some other complications (Lesser, 2000; Smyth et al., 2003). Therefore, it is important to develop therapies that can depress seizures and reduce the epileptic brain injury.

Xenon is used as a safe anesthetic for its fewer side effects. In the recent years, reports of neuroprotective effects of xenon have been attracting attention (Metaxa et al., 2014; Yang et al., 2014; Lavaur et al., 2016). Moreover, xenon can significantly inhibit the uptake and efflux of glutamate (Lavaur et al., 2016), and swiftly terminate the synchronous discharge (Uchida et al., 2012). Over excitation induced by elevated concentration of glutamate is closely associated with epileptic development (Sun et al., 2013) and seizures. Neuronal injury and synchronous discharge are the pathological characteristics of epilepsy (During and Spencer, 1993; Chiu et al., 2016; Kim and Kang, 2018). Therefore, we have a reason to speculate that xenon may possess anti-epileptic properties. Considering that xenon has almost no adverse reactions, xenon treatment may be an effective, and safe intervention for epilepsy.

However, several issues, such as the difference in the anti-seizure effect under different xenon ratio, still need to be addressed for xenon to be a potential clinical intervention. Moreover, as the patients generally could not be treated with xenon immediately, there will be a delayed period between the seizure onset and xenon treatment; therefore, it is also necessary to define the effective therapeutic time-window.

Thus, our study aimed to explore the range of xenon ratio for confirming the most effective ratio for seizure therapy. Furthermore, we investigated the difference in the anti-seizure effect upon xenon treatment at different time points from the seizure onset and establish the effective time-window and the best time-window for xenon treatment. Also, our study preliminarily reveals the underlying mechanisms behind the anti-seizure effect of the xenon mixture.

## Materials and Methods

### Animals and Surgery

Male Sprague-Dawley rats (240–260 g, Certificate No. SCXK2014-0006; Jinan Jinfeng Experimental Animal Co., Ltd, Shandong, China) were used in the study. The experiments were conducted in compliance with the National Institutes of Health *Guide for the Care and Use of Laboratory Animal* (NIH Publications No. 80-23, revised 1996) and the ethical principles of Binzhou Medical University Animal Experimentation Committee (approval no. 2016002). Attempts were made to reduce the number of rats used and their suffering. Experimental rats were maintained in individual cages. Water and food were provided *ad libitum*. All investigations and treatments were carried out between 9:00 and 17:00.

Rats were fixed in a stereotactic apparatus (Anhui Zheng Hua Biological Instrument Equipment Co., Ltd., China) after anesthesia (sodium pentobarbital, 50 mg/kg, i.p., CAS, 57-33-0, Xiya Reagent, China). Each rat was implanted a cannula (RSD Life Science, China) into the lateral ventricle (anteroposterior, AP: −1.8 mm, lateral, L: −1.0 mm, and ventral, V: −3.6 mm). The recording stainless steel electrodes (twisted-pair, A.M. Systems, United States) with 0.5 mm uncoated tip were implanted into the right cortex (AP: −3.2 mm, L: −3.0 mm, and V: −1.8 mm). The implanted electrodes were connected with a microsocket. Both the electrodes and cannulas were fixed onto the skull using dental cement as we have reported previously (Sun et al., 2018). The rats were allowed to recover for 7 days before further experiments.

### KA Treatment and Xenon Inhalation

Kainic acid (KA) was injected stereotaxically (3.25 × 10^–3^ mg/kg, 1.25 mg/ml, Sigma–Aldrich) through a cannula into the lateral ventricle to induce the status epilepticus (SE), as described in our previous study (Zhang et al., 2019). All 126 rats showed continuous generalized seizures almost immediately after the KA injection. The seizures were terminated by diazepam injections (1 mg/ml solution at a dose of 0.002 mg/g body weight; intraperitoneally, Sigma–Aldrich) after 60 min.

Rats were randomly selected and treated with different ratio xenon mixture (Dalian Special Gas Co., Ltd., China, 70% xenon, 21% oxygen, 9% nitrogen treatment, 70% xenon group, *n* = 9; 50% xenon, 21% oxygen, 29% nitrogen treatment, 50% xenon group, *n* = 10; or 35% xenon, 21% oxygen, 44% nitrogen treatment, and 35% xenon group, *n* = 10) based on the previous reports of neuroprotective effects by xenon treatment (Dingley et al., 2006; Cattano et al., 2011). Xenon inhalation was performed just after the KA treatment and lasted for 1 h. The rats in control group (*n* = 10) were treated with 21% oxygen, 79% nitrogen (Rulin Gas Co., Ltd., China) instead of xenon mixture (De Deken et al., 2018). The behavior was monitored for 1 h until the injection of diazepam to terminate the seizures, and electroencephalograms (EEGs) were also recorded and digitalized using filters (1 Hz low-pass and 50 Hz high-pass). Frequency spectrum and the power spectrum density (PSD) in EEGs were analyzed using a PowerLab Biological Recording System (1–50 Hz, AD Instruments, Australia) from KA administration to diazepam injection in each group. In different time point xenon-treatment groups, rats were randomly selected and treated with 70% xenon, 21% oxygen, 9% nitrogen delayed 15 min (xenon 15 min group, *n* = 9) or 30 min (xenon 30 min group, *n* = 8) after the KA treatment. The rats in control groups were treated with 21% oxygen, 79% nitrogen at the same time points (15 min or 30 min, *n* = 10/group). The details of the experimental procedure are shown in Supplementary Figure S1.

The rats received xenon inhalation in a transparent resin observation box with a bottom air inlet and an upper air gate. The gas mixtures were delivered at a same speed (200 ml/min) regulated by a flow regulator valve (DaTe special gas Ltd., China), which was installed in the gas containers. The rat temperature was stable, and the rats were allowed free movement after xenon inhalation.

Seizure severity was assessed using Racine’s criteria (Racine, 1972). Stages 1–3 were considered as focal seizures and stages 4 and 5 were considered as generalized seizures. The placement of cannulas was histologically verified after the experiment. Only the rats with successful implantation in right lateral cerebral ventricle were included in the statistical analysis.

### Fluoro-Jade B (FJB) Staining

Fluoro-Jade B (FJB) is a polyanionic fluorescein derivative, which sensitively and specifically binds to the degenerating neurons (Liu et al., 2018). At the designated time points (24 h, 3 or 7 day after KA administration), 4 rats from each group were deeply anesthetized and perfused intracardially with normal saline and 4% paraformaldehyde in PBS sequentially. Using a cryostat (CM3050s, Leica, Germany), 10-μm thick coronal slices were cut from the extracted brains. Firstly, the tissue slides were immersed in 80% alcohol solution containing 1% sodium for 5 min followed by 70% alcohol for 2 min and distilled water for 2 min. Then, in order to ensure the same background between the slides, the slides were immersed in a solution containing 0.06% potassium permanganate for 15 min on a shaking rocker; finally, the slides were rinsed for 2 min in distilled water. The FJB dye powder was used to prepare a 0.01% stock solution. Staining solution was made with 96 ml 0.1% acetic acid and 4 ml FJB stock solution, and was used within 10 min of preparation. The slides were immersed in the staining solution for 20 min and were rinsed for 1 min in distilled water. The slides were then placed in the oven at 50°C for 5 min. Lastly, the slides were immersed in xylene for 1 min and were mounted by neutral balsam and coverslipped with DPX (Sigma, United States). The slides were observed under the epifluorescent microscope (CX41, Olympus, Japan) with blue (495 nm) excitation filter. Positive signals observed in the slides were counted manually.

### Statistical Analysis

All data were acquired in a blinded manner and presented as mean ± SEM. Statistical analysis was carried out by SPSS v16.0 (SPSS Inc., Chicago, IL, United States) for Windows. The non-parametric Mann-Whitney *U*-test was used to analyze the cumulative time spent in each seizure stage and the seizure stage at different time points after KA administration. One-way ANOVA with Dunnett’s T3 *post hoc* test was used to analyze the effect of different ratio xenon mixtures on cumulative seizure duration and cumulative generalized seizure duration (GSD). Positive signals of FJB staining were also analyzed by one-way ANOVA with Dunnett’s T3 *post hoc* test. One-way ANOVA was used to assess the effect of xenon inhalation at different time points (15 or 30 min) on seizure duration and GSD as compared with the controls. For all analyses, a *P* < 0.05 was considered as significant.

## Results

### The Effect of Different Ratio Xenon Mixture Inhalation on KA-Induced SE

**FIGURE 1:**
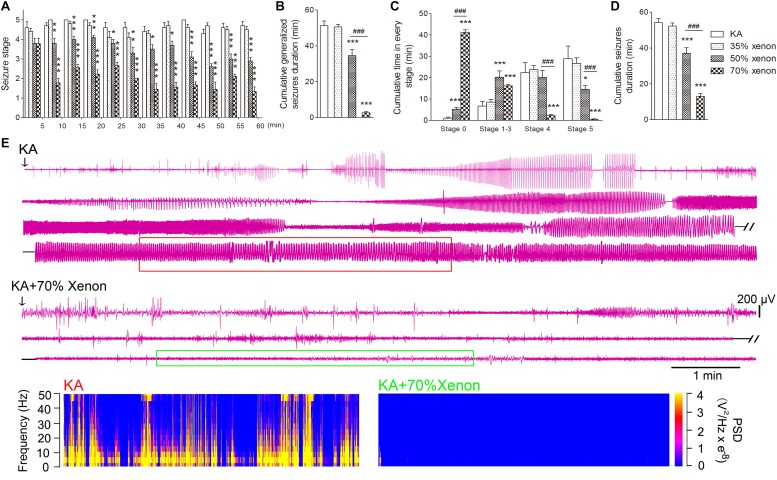
The effect of different ratio xenon mixtures inhalation on the KA-induced SE. **(A)** The change in seizure stage after KA administration. **(B)** Cumulative generalized seizures duration (GSD). **(C)** Cumulative time in each stage. **(D)** Cumulative seizures duration. **(E)** Representative EEGs, frequency spectrum, and the power spectrum density (PSD) changes from each group. KA group, *n* = 10; 35% xenon group, *n* = 10; 50% xenon group, *n* = 10; and 50% xenon group, *n* = 9. Means ± SEM are shown. The non-parametric Mann-Whitney *U* test was used to analyze the cumulative time spent in each seizure stage and the seizure stage at different time points after KA administration. One-way ANOVA with Dunnett’s T3 *post hoc* test was used to analyze the effect of different ratio xenon mixtures on cumulative seizure duration and cumulative GSD. ^*^P < 0.05, ^∗∗^*P* < 0.01, and ^∗∗∗^*P* < 0.001 vs. controls. ^###^*P* < 0.001 compared with each other.

The different ratio xenon mixtures (70% xenon, 21% oxygen, 9% nitrogen, 50% xenon, 21% oxygen, 29% nitrogen, 35% xenon, 21% oxygen, and 44% nitrogen) were inhaled immediately after KA treatment for 1 h. The investigation lasted until the seizures were terminated by diazepam injections after 1 h. The rats in 50 or 70% xenon inhalation groups showed significantly attenuated seizure stage from the second 5 min onward as compared with the control group treated with 21% oxygen, 79% nitrogen (*P* = 0.006 and *P* < 0.001, respectively, Figure 1A). The seizure stages in each group were evaluated further. The results showed that the cumulative generalized seizures duration (in stages 4 and 5) was significantly reduced after 50 or 70% xenon treatment (KA, 53.3 min; 50% xenon, 34.6 min; 70% xenon, 3.4 min; *P* < 0.001 and 0.001, Figure 1B). Further analysis showed that 70% xenon treatment significantly prolonged the cumulative time in stage 0 (no epileptic seizures, KA group, 0 min; 70% xenon group, 44.1 min; *P* < 0.001, Figure 1C), but reduced the time spent in stage 4 and 5 (*P* < 0.001 and 0.001, Figure 1C). Moreover, the rats in 50% xenon treated group also spent more time in stage 0 (*P* < 0.001, Figure 1C) and focal seizures (stages 1–3, *P* < 0.001, Figure 1C), but shorter time in generalized seizures (*P* < 0.001, Figure 1B). EEGs were recorded after KA administration for 60 min until the diazepam injection. The cumulative seizure duration was 13.0 min in 70% xenon mixture group and 54.3 min in the control group (*P* < 0.001, Figure 1D). The cumulative time in stages 4 and 5, as well as seizure duration in 70% xenon treated group were less than the 50% xenon treated group (*P* < 0.001, Figure 1B). The representative EEGs, frequency spectrum, and the PSD changes from each group are shown in Figure 1E. The behavioral and EEG results indicate the strong inhibitive effect of 50 and 70% xenon in KA-induced epileptic seizures. 70% xenon mixture led to a stronger anti-seizure effect, while no significant effect was observed in rats treated with 35% xenon.

### The Effect of Different Ratio Xenon Mixture Inhalation on KA-Induced Neuronal Degeneration

**FIGURE 2:**
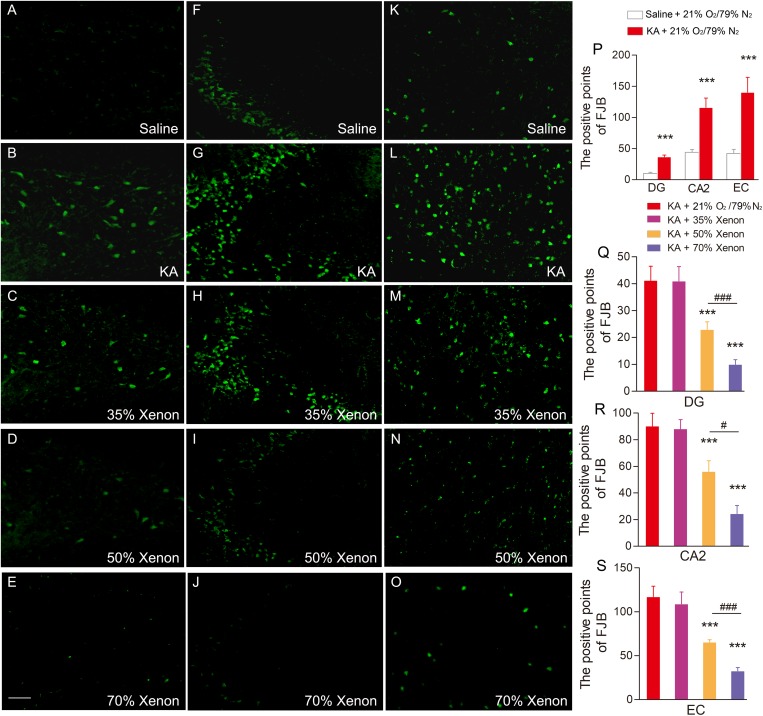
The effect of different ratio xenon mixture inhalation on the KA-induced neuronal degeneration on day 7, 50 and 70%, but not 35% xenon mixture attenuated the neurodegeneration in the hippocampus (**A–E**, dentate gyrus; **F–J**, CA2), and EC **(K–O)** induced by KA administration (*n* = 4 per group; bar = 50 μm). **(P–S)** Quantified positive signals of FJB staining. Means ± SEM are shown. One-way ANOVA with Dunnett’s T3 *post hoc* test was used to analyze the effect of different ratio xenon mixtures on positive signals of FJB staining. ^∗∗∗^*P* < 0.001 vs. controls. ^#^*P* < 0.05 and^###^ *P* < 0.001 compared with each other.

Fluoro-Jade B, a dye that sensitively and specifically binds to the degenerating neurons, was used to analyze the degenerating neurons in different groups. The positive signals for FJB were found to be increased in the hippocampus and entorhinal cortex (EC) at all the investigated time points (24 h, 3 day, and 7 day) after KA administration. The representative increased FJB signals on day 7 are shown in Figure 2 (dentate gyrus, *P* < 0.001; CA2, *P* < 0.001; Figures 2B,G,P) and entorhinal cortex (EC, *P* < 0.001, Figures 2L,P), as compared to the rats treated with saline. However, the increase in the FJB positive staining was attenuated in the 50 and 70% xenon treated groups (Figures 2D,E,I,J,N,O,Q–S) and 70% xenon treatment provided a stronger inhibitory effect as compared to the 50% xenon treatment (dentate gyrus, *P* < 0.001, Figure 2Q; CA2, *P* = 0.019, Figure 2R; EC, *P* < 0.001, Figure 2S). With an increase in the ratio of xenon, the FJB positive signal reduced (Figures 2C–E,H–J,M–O,Q–S). There was no significant difference between the 35% xenon group and the saline group (Figures 2C,H,M,Q–S).

### The Time-Window Effect of 70% Xenon Mixture Inhalation Delayed by 15 min or 30 min on the KA-Induced SE

**FIGURE 3:**
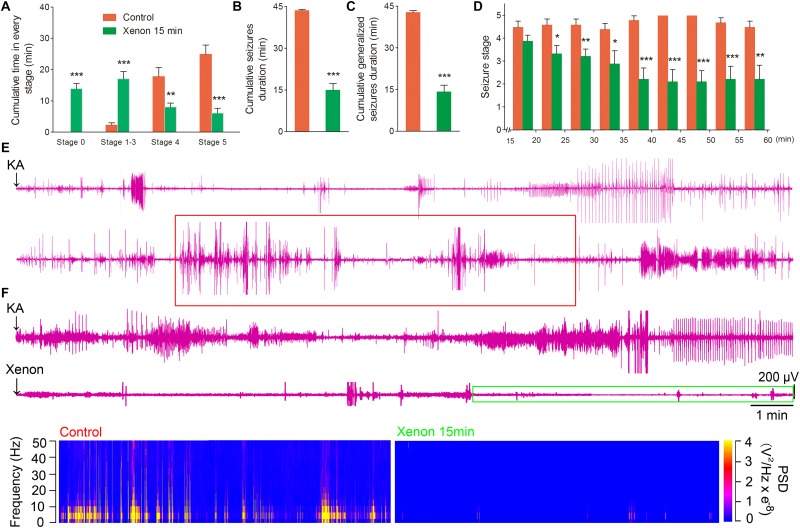
The time-window effect of 70% xenon mixture inhalation delayed by 15 min on the KA-induced SE. Delayed 15 min treatment of 70% xenon mixture (*n* = 9) significantly prolonged the cumulative time in stages 0–3 and reduced the cumulative time in stages 4 and 5 **(A)**, cumulative seizures duration **(B)**, cumulative GSD **(C)**, and attenuated seizure stage from second 5 min onward after xenon treatment **(D)**, compared with the control group (*n* = 10). **(E**,**F)** Representative EEGs, frequency spectrum, and the PSD changes. Means ± SEM are shown. One-way ANOVA was used to assess the role of xenon inhalation at different time points (0 min or delayed 15 min). ^*^P < 0.05, ^∗∗^*P* < 0.01, and ^∗∗∗^*P* < 0.001 vs. controls.

**FIGURE 4:**
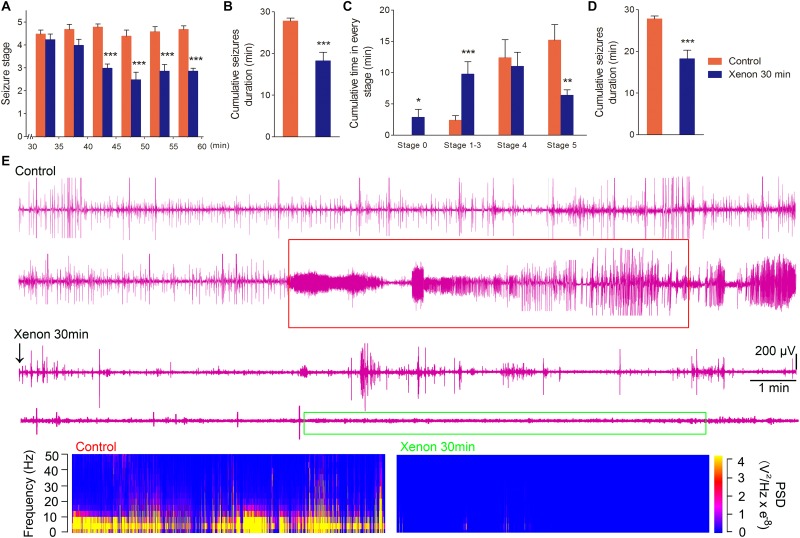
The time-window effect of 70% xenon mixture inhalation delayed by 30 min on the KA-induced SE. Delayed 30 min treatment of 70% xenon mixture (*n* = 8) provided significant anti-seizure effect from the third 5 min after xenon treatment **(A)**, reduced cumulative seizure duration **(B)**, cumulative time in stages 4 and 5 **(C)**, prolonged the cumulative time in stages 0–3 **(C)**, and cumulative GSD **(D)** compared with the control group (*n* = 10). Representative EEGs, frequency spectrum, and the PSD changes. **(E)** Means ± SEM are shown. One-way ANOVA was used to assess the role of xenon inhalation at different time points (0 min or delayed 30 min). ^*^P < 0.05, ^∗∗^*P* < 0.01, and ^∗∗∗^*P* < 0.001 vs. controls.

To evaluate the appropriate time window of xenon treatment to inhibit seizures, 70% xenon mixture was inhaled from 15 min or 30 min after KA administration, and the inhalation lasted for 45 min or 30 min, respectively, until the diazepam was injected to terminate the seizures. The behavioral results showed that the rats in xenon 15 min group still significantly prolonged the cumulative time in stages 1–3 (*P* < 0.001, Figure 3A) and had no behavioral seizures (stage 0, *P* < 0.001, Figure 3A), while the cumulative time in generalized seizures (stages 4 and 5) was significantly reduced (Figures 3A,C). During the xenon inhalation period, the stage of seizures was significantly attenuated from the second 5 min onward in the xenon 15 min group (*P* = 0.012, Figure 3D). The average seizure stage at 60 min was 2.2 in the xenon 15 min group and 4.5 in the control group (*P* = 0.003, Figure 3D). The reduced cumulative seizure duration was also observed in the xenon 15 min group as compared to the control (43.5 and 14.9 min, *P* < 0.001, Figure 3B). The similar inhibitive effect was also observed in xenon 30 min group, such as attenuated seizure stage (*P* < 0.001, Figure 4A), prolonged cumulative time in stage 0 (*P* = 0.02) and stages 1–3 (*P* < 0.001, Figure 4C), reduced cumulative time in generalized seizure (*P* < 0.001, Figure 4D), and cumulative seizure duration (*P* < 0.001, Figure 4B). The represented EEGs, frequency spectrum, and the PSD changes are shown in Figures 3E,F, 4E. The results indicate the significant inhibitive effect of 70% xenon treatment delayed for 30 min after the epileptic seizures.

### The Effect of 70% Xenon Mixture Inhalation Delayed by 15 min or 30 min on KA-Induced Neuronal Degeneration

**FIGURE 5:**
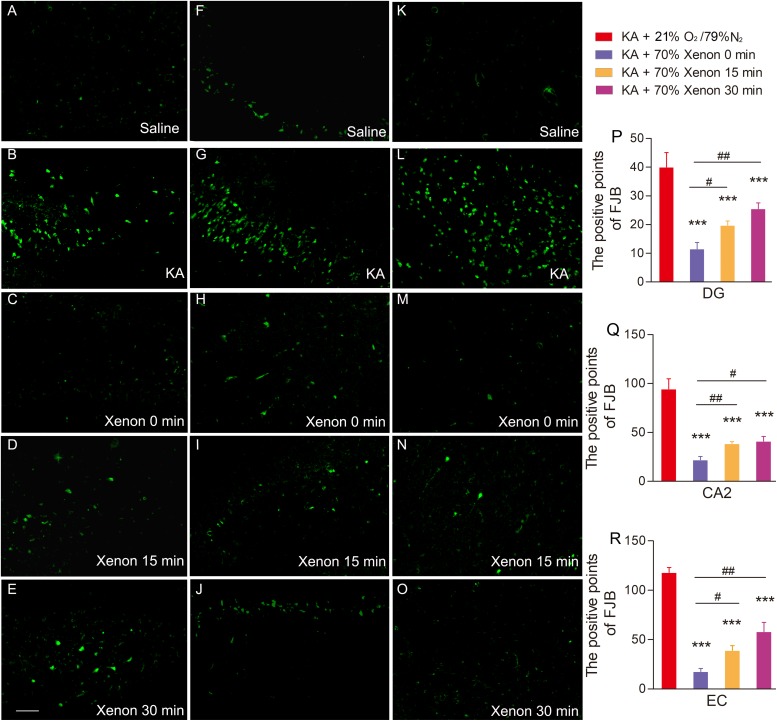
The effect of 70% xenon mixture inhalation delayed by 15 or 30 min on the KA-induced neuronal degeneration. **(A–O)** Different effects of xenon inhalation immediately (delayed 0 min), delayed by 15 min or delayed by 30 min on neurodegeneration induced by KA administration (dentate gyrus, **A–E**; CA2, **F–J**; EC, **K–O**; *n* = 4 per group; bar = 50 μm). **(P–R)** Quantified positive signals of FJB staining. Means ± SEM are shown. One-way ANOVA with Dunnett’s T3 *post hoc* test was used to analyze the neuroprotective effect of xenon treatment at different time points. ^∗∗∗^*P* < 0.001 vs. controls. ^#^*P* < 0.05, ^##^*P* < 0.01, and ^###^ *P* < 0.001 compared with each other.

The FJB staining was performed on day 7 in all the groups: saline group, KA group, and xenon inhaled groups treated at different time points after KA administration (immediately, 15 min, and 30 min). The results showed that 70% xenon mixture treatment noticeably reduced the FJB positive signals, even after delayed for 30 min, compared with the KA group treated with 21% oxygen, 79% nitrogen, in the hippocampus (dentate gyrus, *P* < 0.001, Figures 5A–E,P; CA2, *P* < 0.001, Figures 5F–J,Q), and EC (*P* < 0.001, Figures 5K–O,R). However, with the delay in treatment, the inhibitive effect of xenon mixture was found to be attenuated (Figures 5C–E,H–J,M–O,P–R).

## Discussion

Our study confirmed the significant therapeutic effect of xenon inhalation on the KA-induced SE. Moreover, we evaluated the therapeutic effect of different proportional xenon gradient at various delayed time-points. We found that the anti-seizure effect is closely associated with the proportion of xenon. The significant therapeutic effect was found in 70% and 50% xenon mixture treated group, but not in 35% xenon treated group. Additionally, both 15 min- and 30 min-delayed treatment displayed a significant therapeutic effect.

Xenon, an inert gas that has been used in the clinics, hardly participates in any chemical reaction and does not undergo biological transformation *in vivo*. It is exhaled through the lungs in its original form after inhalation. It has been characterized with non-toxic side effects and high safety. On the other hand, the current anti-epileptic therapies have noticeable problems, such as the serious adverse reactions and drug resistant of anti-epileptic drugs (Schmidt and Löscher, 2005; Chen et al., 2018). Consequently, xenon treatment may possess good and safer prospects as the epilepsy intervention in the clinics.

Previous studies have provided sufficient evidence for the neuroprotective effects of xenon such as attenuation of the neuronal injury, apoptosis, and neuronal loss in several neuronal diseases, such as Alzheimer’s disease (Lavaur et al., 2016), unilateral common carotid artery ligation (Metaxa et al., 2014), intrauterine asphyxia (Yang et al., 2012), and neonatal asphyxia (Luo et al., 2008). Furthermore, it has been reported that xenon could effectively terminate the synchronous discharge by suppressing the glutamate intake and release (Uchida et al., 2012). Excessive glutamate-induced excitatory toxicity is an important mechanism in epileptic seizures and propagation (During and Spencer, 1993; Chiu et al., 2016; Goubert et al., 2017; Luna-Munguia et al., 2019). Extracellular glutamate accumulation can over-stimulate the corresponding receptors such as N-methyl-D-aspartate (NMDA) and α-amino-3-hydroxy-5-methyl-4-isoxazolepropionic acid (AMPA) receptors, and over-excitation of the NMDA receptors plays an imperative role in the occurrence and development of acute nerve injury. It can eventually lead to neuronal dysfunction and apoptosis by activating the calpain and caspase-3 pathways (Baudry and Bi, 2016; Hoque et al., 2016; Izumida et al., 2017; Deng et al., 2019). On the other hand, synchronous discharge, neuronal injury, and even neuronal loss are the typical electroencephalogram and pathological features of epilepsy (Aracri et al., 2018; Bumanglag and Sloviter, 2018; Chang et al., 2018). For example, apoptosis is known to be involved in the formation of hippocampal sclerosis in the patients with medial temporal lobe epilepsy (Danis et al., 2016). Apoptosis and degeneration of the hippocampal neurons have been observed in the animal model of temporal lobe epilepsy induced by KA administration. In the model of acute temporal lobe epilepsy induced by KA injection into the hippocampal CA3 region, the caspase-3 pathway was activated, which further induced the hippocampal neuron apoptosis (Liang et al., 2016). The slow kindling epilepsy model induced by intraperitoneal injection of pentylenetetrazole (PTZ) was accompanied by the cortical neuron apoptosis (Al-Shorbagy and Nassar, 2017). The anticonvulsant effect by subanesthetic levels of xenon was observed in neonatal asphyxial seizures (Azzopardi et al., 2013). The previous reports, thus, strongly suggest that xenon inhalation may possess a therapeutic role in both epileptic seizures and epilepsy-induced neuronal injury. Additionally, our study further confirms the therapeutic effect of xenon mixture inhalation on the KA-induced SE as well as the neuronal injury.

The therapeutic effects of any drug or treatment are commonly associated with the dose administration. It is well known that too small dose is ineffective and too large dose can lead to toxic effects. Different doses can sometimes even lead to opposite effects. Consequently, the effective range of xenon ratio for the best anti-seizure effect needs to be well-defined. Our study confirms the anti-seizure effect and attenuated neurodegeneration by 50 and 70% xenon mixtures. Moreover, the therapeutic effect of 70% xenon mixture was found to be significantly stronger than the 50% xenon mixture.

Delayed therapeutic period between the seizure onset and the treatment is unavoidable in the clinics. Delayed treatment is often different from immediate drug administration and also from other therapeutic methods such as deep brain stimulation (Wang et al., 2008; Wu et al., 2008). Consequently, it is meaningful to define the effective time-window of xenon treatment. Seventy percent xenon mixture, which displayed the strongest effects in our study, was inhaled at different time points after KA administration. Our study indicates that the 70% xenon mixture treatment displayed the anti-seizure effect at all the three time points (0, delayed 15, and delayed 30 min) after KA administration and reduced the extent of neurodegeneration. The results confirm the therapeutic effects of xenon inhalation delayed by less than 30 min after the onset of seizures. These results are meaningful for the possible clinical applications in the future.

Neuronal injury, such as neurodegeneration and apoptosis, are the prominent pathological features of epilepsy in both animal models and human patients (Bumanglag and Sloviter, 2018; Chang et al., 2018). FJB staining was used to evaluate the neuroprotective effects in our study because FJB sensitively and specifically binds to the degenerating neurons (Liu et al., 2018). Our study demonstrated increased FJB positive signals in the hippocampus and EC in KA-induced SE. Moreover, the increased neurodegeneration was reversed after the treatment with appropriate xenon ratio (50 or 70%) and proper delayed time periods (0–30 min), consistent with the effective anti-seizure parameter. The results indicate that the neuroprotective effects of xenon mixture may contribute toward its anti-seizure characteristic.

The FJB staining confirmed the neurodegeneration in the hippocampus and EC, which are the vital brain subregions in the epileptic network (Hsu, 2007; Xu et al., 2010). Hippocampus is considered as the “promoter” or “amplifier” in the epileptic network (Heinemann et al., 1992; Hsu, 2007). The hippocampus was reported to be the primary driving region for the seizures and the pathway for both longitudinal (Derchansky et al., 2006) and contralateral (Blackstad, 1956) epileptiform activity propagation. Several epileptic animal models have shown that a defect in the hippocampus is vital for epileptogenesis (Heinemann et al., 1992; Hsu, 2007). Moreover, the hippocampal CA3 region emits low frequency discharge, which could depress the epileptiform activity generated from the EC, thus, inhibit the activation of CA1-subiculum networks (Barbarosie and Avoli, 1997). The EC, on the other hand, is considered as a gateway to the hippocampus and, similar to the hippocampus, plays a vital role in the development of epilepsy and epileptic seizures (Xu et al., 2010). A remarkable depress network in the EC has been reported during the transition to a seizure (Gnatkovsky et al., 2008). The interictal-like discharges of EC could attenuate the epileptic synchronized activity in the limbic networks (Barbarosie and Avoli, 1997; D’Arcangelo et al., 2005).

These studies indicate that the integrity of the hippocampal and EC neurons may be the vital regulating point in epilepsy. Our research provides the evidence for KA-induced neuronal injury in the hippocampus and EC. The defect in the hippocampal and EC neurons may lead to loss of the effective control over depressed network. Moreover, the results also confirm that an appropriate model of xenon treatment could inhibit seizures and reverse the hippocampal and EC neuronal injury synchronously.

In summary, our study confirms the anti-seizure effect of xenon mixture in the KA-induced SE. Moreover, we optimized the effective xenon ratio and appropriate time-window of therapeutic intervention. The xenon therapeutic effect may be produced by attenuation of the hippocampal and EC neuronal injury and should be further explored as a potential intervention for seizures and epilepsy.

## Data Availability

All datasets generated for this study are included in the manuscript and/or the Supplementary Files.

## Ethics Statement

This study was carried out in accordance with the recommendations of “The National Institutes of Health Guide for the Care and Use of Laboratory Animal (NIH Publications No. 80-23, revised 1996).” The protocol was approved by the “Binzhou Medical University Animal Experimentation Committee (approval no. 2016002).”

## Author Contributions

YZ and MZ: study conception and design and data acquisition. JY, WZ, and QW: KA-induced rat model preparation and xenon treatment. XP, XG, and JY: data acquisition and data analysis. HS: study design, data acquisition, and drafting of the manuscript.

## Conflict of Interest Statement

The authors declare that the research was conducted in the absence of any commercial or financial relationships that could be construed as a potential conflict of interest.

## Supplementary Material

FIGURE S1 The details of the experimental procedure.
