# Supplementary figures and images for: Mode-Dependent Effect of Xenon Inhalation on Kainic Acid-Induced Status Epilepticus in Rats
Source: Front Cell Neurosci. 2019 Aug 14;13:375. doi: 10.3389/fncel.2019.00375 (PMC6702968; doi:10.3389/fncel.2019.00375)

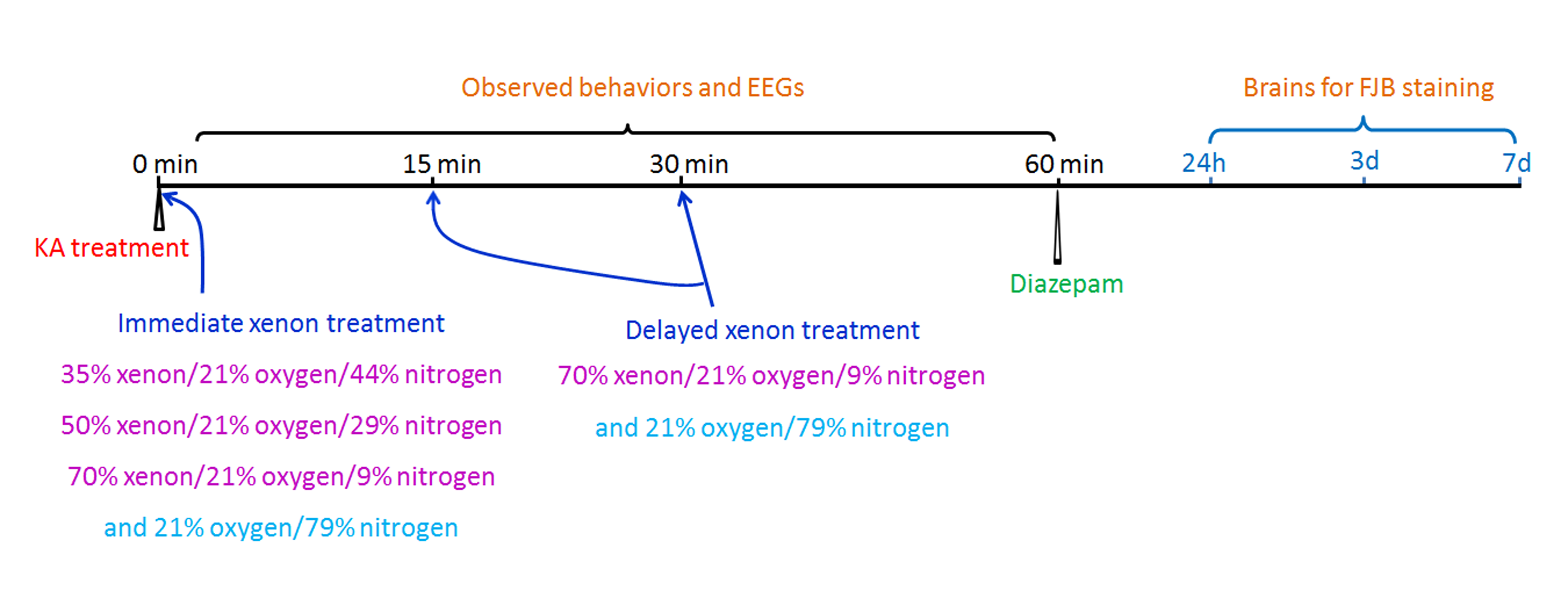

Supplement: FIGURE S1 — The details of the experimental procedure. [file Image_1.TIF]
